# Effects of biocholine powder supplementation in ewe lambs: Growth, rumen fermentation, antioxidant status, and metabolism

**DOI:** 10.1016/j.btre.2020.e00580

**Published:** 2020-12-24

**Authors:** Karoline W. Leal, Davi F. Alba, Marily G. Cunha, Hiam Marcon, Fernanda C. Oliveira, Roger Wagner, Anielen D. Silva, Thalison F. Lopes, Loren S.B. de Jesus, Maria Rosa C. Schetinger, Claiton A. Zotti, Julcemar D. Kessler, Marcelo Vedovatto, Aleksandro S. Da Silva

**Affiliations:** a Postgraduate Program in Animal Science, State University of Santa Catarina (UDESC), Chapecó, SC, Brazil; b Department of Food Science Technology, Federal University Santa Maria (UFSM), Santa Maria, RS, Brazil; c Department of Biochemistry and Molecular Biology, UFSM, Santa Maria, RS, Brazil; d University of the West of Santa Catarina, Xanxerê, SC, Brazil; e Department of Animal Science, UDESC, Chapecó, SC, Brazil; f State University of Mato Grosso Do Sul, Aquidauana, MS, Brazil

**Keywords:** Choline, Nutrition, Sheep, Supplements, Nutritional biotechnology

## Abstract

The objective of this study was to evaluate the effects of biocholine powder (BP) added to the concentrate of ewe lambs on growth, rumen fermentation, antioxidant status, and metabolism. Forty-eight Lacaune lambs were divided 3 treatments: T0, T4, and T8, representing 0, 4, and 8 g of BP per animal/day added to the concentrate, respectively. The T4 and T8 lambs had greater average daily gain from d 0–75 (0.22, 0.25, and 0.26 kg/d for T0, T4 and T8, respectively). T4 and T8 lambs had higher methylene blue test results (indicating microbiological activity), and the higher effects were detected for T8 lambs. The BP in lamb’s diet increased the ruminal concentration of propionate (T4 and T8). The T8 lambs had greater serum activity of superoxide dismutase and glutathione transferase, and lower reactive oxygen species levels. We conclude that BP is a biotechnological additive with high nutritional potential.

## Highlights

• Supplementation with biocholine powder (BP) had no effect on the performance in young female sheep to long-term.
• But supplementation with BP in the first 30 days of ingestion potentiates weight gain.
• Ingestion of BP modified the ruminal fermentation profile, and decreased the concentration of volatile fatty acids.
• BP supplementation stimulated antioxidant, immunological and hepatoprotective activities.

## 1 Introduction

Lamb rearing systems have direct impacts on parameters related to reproductive efficiency. On milk production farms, intensive feeding systems of young animals ensure their growth and development, as these animals require better care. Simplício and Maia [1] reported that body development and the age of puberty in lambs depend on the environment, genetics and gender; nevertheless, these events are strongly influenced by the production system, nutrition, and feed.

Females who enter puberty before achieving minimum body weight may have reduced body development, resulting in smaller offspring. Simplício and Maia [1] pointed out that this happens when management, feeding, nutrition, and health care are deficient. In dairy sheep production systems, is normal provide ewes milk for the lambs between days 2–5 of age, and then milk replacer until 50–60 days of age, when they are weaning [2]. Therefore, it is necessary, in addition to providing appropriate feeding systems, to use strategic supplementation to enhance animal zootechnical performance. According to Silva Sobrinho [3], on most farms, there are deficiencies of essential nutrients attributable to the lack of commercial products formulated specifically for sheep. For these reasons, it is important to provide supplements so as to minimize these negative effects. Researchers have suggested, for example, supplementation with minerals, vitamins or herbal extracts [4].

Silva Sobrinho [3] suggested that dietary supplements may alter the digestibility and/or the consumption of roughage. An important ingredient in animal diet is choline, generally available to animals as choline chloride [5]. Choline is essential for maintenance of various physiological functions, including metabolism, maintenance and cell integrity [6]. Deficiencies contribute to heart disease, growth, and bone development abnormalities, as well as compromised liver and kidney functions [7]. For ruminants, choline is degraded in the rumen; therefore, it is necessary to provide rumen-protected choline [8,9].

The bioavailability of choline is influenced by dose, mode of administration, stage of animal development, and dietary composition [10]. In concentrate, choline is present in the form of lecithin; in compound and commercial feed, choline chloride is added as an alternative, but is easily degraded, resulting in low bioavailability for intestinal absorption [10]. Recent studies have shown good results with biocholine powder [[11], [12], [13], [14]].

Vegetable biocholine (VB) or biocholine powder (BP) is extracted from plants; it has natural resistance to ruminal degradation in the form of phosphatidylcholine [15]. Nevertheless, information about its ruminal fermentation is scarce. In the 1970s, researchers observed that choline chloride was rapidly metabolized, leaving doubts as to its true influence on bacterial growth mechanisms [16]. BP needs to be further explored, especially related to the effects on rumen fermentation and short and/or long-term effects on growth performance. Therefore, the objective of this study was to determine the effects of BP on growth, rumen fermentation, antioxidant status, and metabolism of ewe lambs.

## 2 Materials and methods

### 2.1 Biocholine

Biocholine Powder® (Nutriquest, Brazil) is the trade name of a phosphatidylcholine product, a molecule extracted from the plants *Azadirachta indica*, *Citrullus colocynthis*, *Trachyspermum ammi* and *Achyranthes aspera*. A sample of this additive was sent to a specialized laboratory for actual quantification of total phospholipids and phosphatidylcholine in BP using high-performance thin-layer chromatography [17]; we found levels of total phospholipids of 16.8 g/kg and 9.74 g/kg of phosphatidylcholine.

The chemical composition of the commercial product, biocholine powder, was chemically evaluated: 92.6 % dry matter, 9.77 % crude protein, 5.72 % ether extract, 36.4 % acid detergent fiber, and 45.1 % neutral detergent fiber.

### 2.2 Animals and experimental design

The experiment was carried out in a dairy farm located in Chapecó, SC, Brazil after approval by the Animal Experimentation Ethics Committee (CEUA/UDESC), protocol 8560130319.

Forty-eight Lacaune lambs [females; 4 months of age; 28.15 ± 3.83 kg of body weight (BW)] were stratified by BW and then assigned to 12 pens (four lambs/pen); corresponding to three treatments, with four repetitions. All animals were clinically evaluated and were apparently healthy.

The pens were located in a covered shed with a slatted floor. Each pen had one drinking fountain and two feed bunk, the concentrate being offered to the animals once a day (morning) (400 g/animal/day; Table 1); the concentrate was entirety consumed by the four animals within 15 min after delivery. The silage was provided twice a day (0700 h and 1700 h), divided equally and offered daily in the amount of 4.5 % of body weight. Daily silage consumption was not measured, but it was observed that there were leftovers of approximately 10%–15% of the supplied/day. As the experiment was carried out on a commercial farm, the diet (feed formulation and quantity provided) was not changed, only the BP was added to the concentrate.

**Table 1:** Ingredients and chemical composition of feeds used in the study.

Ingredients		Natural matter (kg/animal/day)			Dry matter (kg/animal/day)	
Corn silage		2.00			0.66	
Concentrate^a^		0.40			0.35	
Chemical composition^b^	Corn silage		Concentrates			
			T0	T4		T8
Dry matter (DM), %	32.90		88.50	88.50		88.90
*% of DM*						
Crude protein	8.01		15.90	15.91		15.81
Ash	4.21		5.63	5.84		6.38
Ether extract	4.32		4.20	4.40		4.20
NDF	33.00		8.64	10.50		9.83
ADF	17.80		3.41	4.87		4.88
TDN^c^	73.95		81.66	81.27		80.52
*Mcal/kg of DM*						
NEm^d^	1.76		1.99	1.98		1.96
NEg^d^	1.14		1.34	1.33		1.31

a Total 100 kg of base concentrate was formulated with corn (70 %), soybean meal (25 %) and premix (5%). The premix used in this study contained phosphorus (min 55 g/kg), calcium (215 g/kg, max225 g/kg), sulfur (min 12 g/kg), sodium (min 80 g/kg), cobalt (min 60 mg/kg) chromium (min 12 mg/kg), iron (1420 mg/kg), iodine (min 14 mg/kg), magnesium (min 14 mg/kg), manganese (min 1550 mg/kg), selenium (min 22 mg/kg), vitamin A (2000 IU/kg), vitamin D (min 40,000 IU/kg), vitamin E (min 550 IU/kg) and fluorine (max550 mg/kg).

b NDF (neutral detergent fiber), ADF (acid detergent fiber), TDN (total digestible nutrients), NEm (net energy for maintenance) and NEg (net energy for gain).

c Calculated as described by Weiss et al. [20].

d Calculated using the equations proposed by the NRC [21].

The pens were randomly assigned to the treatments (four pen/treatment): T0, T4, and T8, representing 0, 4, and 8 g of BP per animal/day, respectively. The treatments were added in the concentrate at 0, 10, and 20 g/kg, representing 0, 4, and 8 g of BP per animal/day, respectively. The doses used in this study were based on the manufacturer's recommendation and also on previous studies from our research group [18].

### 2.3 Analysis of the chemical composition of feed

Silage and concentrate were sampled, frozen, and analyzed for chemical composition (Table 1). The feeds were analyzed according to AOAC [19]: dry matter (DM), method 930.15; crude protein (CP), method 976.05; ether extract (EE), method 920.39 and ash, method 942.05. The concentration of neutral detergent fiber (NDF) and acid detergent fiber (ADF) was measured according to the methodology of Van Soest et al. (1991) without the addition of sodium sulfite or alpha-amylase. The concentrations of total digestible nutrients (TDN) were calculated according to Weiss et al. [20] and the net energy for maintenance (NEm) and gain (NEg) were calculated according to NRC [21].

### 2.4 Growth performance

The BW was evaluated on d 0, 15, 30, 45, 60, and 75 of the experiment. All animals were weighed individually using an analytical digital scale.

### 2.5 Blood and ruminal fluid collection

Blood samples were collected from jugular veins of eight animals per treatment, using vacuum tubes without anticoagulant to obtain serum on d 0, 15, 30, 45, 60, and 75 of the experiment. Immediately after collection, the tubes were refrigerated at 10 °C, then centrifuged at 3800 × *g* for 10 min. The serum was collected, identified, and stored at −20 °C until analysis.

Ruminal fluid was collected two hours after the morning feeding on d 15, 45, and 75 using a 1.5-m, 11-mm diameter oro-rumen tube. The first 50 mL were discarded for possible salivary contamination, as previously described [[22], [23], [24]]. A total of 200 mL of ruminal fluid was collected per animal, stored in two 200 mL glass Becker® containers. Then, the pH was measured using a portable digital pH meter, model AK103. One part of the sample was used for the methylene blue reduction test (MBT) to identify microbial activity [22].

The remaining ruminal fluid was filtered through three gauze and stored in two 50 mL Falcon® tubes per sample in a thermal box, previously heated with water at 39 °C. The tubes were then frozen at −20 °C for further analysis of volatile fatty acids (VFA).

### 2.6 Analysis of ruminal volatile fatty acids

The VFA concentrations of acetate, propionate and butyrate were determined in ruminal fluid. After thawing in a water bath (final temperature 10 °C), samples were centrifuged at 1050 × *g* for 5 min. Then, 1 mL of supernatants was transferred to 2 mL polypropylene tubes and added 100 μL of citric acid (1.0 mol/L). Subsequently, the contents were homogenized for 30 s in a vortex shaker, followed by centrifugation for 5 min at 17,000 × *g*. Then, 100 μL of supernatants were diluted in 900 μL of methanol and added to 100 μL of an ethyl hexanoate internal standard (8.7 mg/mL in methanol).

Samples were analyzed in a gas chromatograph equipped with a flame ionization detector (GC-FID; Varian Star 3400CX, Chrompack, Middelburg) and an auto sampler system. A total of 1 μL of extract were injected in a split/splitless injector operated in splitless mode for 1 min (the splitter-valve was open 20:1). Hydrogen was used as a carrier gas, at a constant pressure of 25 psi. Analyte separation was carried out on a capillary column CP-WAX 52 CB (60 m ×0.25 mm ×0.25 μm). The initial temperature was adjusted to 50 °C for 1 min, and increase up to 185 °C at 15 °C/min, and increasing at 5 °C/min until 195 °C. Injector and detector temperatures were constant at 240 °C.

Method validation was carried out according to the Eurachem Guide [25] with the following parameters: selectivity, linearity, linear range, repeatability, accuracy and limited of detection (LOD) and limit of quantification (LOQ). Linearity was evaluated by calculating a regression equation using the least-squares method. The analytical calibration curves are displayed Supplementary Material 1. The LOD and LOQ values were obtained by sequential dilution until obtaining signal-to-noise ratios of 3:1 and 10:1, respectively. Precision was assessed by evaluating the repeatability of six replicate analyses. Accuracy was determined by recovering known amounts of standard substances added to the samples. The results were expressed as mmol of each SFA per 100 mL rumen fluid.

### 2.7 Serum clinical biochemistry

Serum levels of total protein (TP), albumin, glucose and urea were quantified using a Bio-2000 semi-automatic analyzer (BioPlus®) and commercial kits (Analisa®). Globulin values were calculated as the difference between TP and albumin. Serum levels of aspartate aminotransferase (AST), alanine aminotransferase (ALT) and gamma glutamyltransferase (GGT) were measured using commercial kits for the Bio-2000.

### 2.8 Oxidant status and antioxidant enzymes

Glutathione S-transferase (GST) activity was assayed spectrophotometrically at 340 nm using the method of Habig et al. [26]. The mixture contained serum as test, 0.1 M potassium phosphate buffer (pH 7.4), 100 mM GSH and 100 mM CDNB, which was used as the substrate. The enzymatic activity was expressed as μmol CDNB/min/mg protein.

The antioxidant enzyme superoxide dismutase (SOD) activity was assayed in serum by measuring the inhibition of 1 mM adrenaline auto-oxidation by absorbance at 480 nm using a glycine buffer (50 mM, pH 10.2) as described by Bannister and Calabrese [27].

Serum ROS levels were determined using the DCF oxidation method described by LeBel et al. [28]. Fluorescence was measured using excitation and emission wavelengths of 485 nm; and a calibration curve was established using 2′,7′-dichlorofluorescein (DCF; 0.1 nM to 1 μM), and results were expressed as U DCF/mg of protein.

### 2.9 Statistical analysis

All dependent variables were tested for normality using the Univariate procedure in SAS (SAS Inst. Inc., Cary, NC, USA; version 9.4) and all variables were normally distributed. All data were then analyzed using MIXED procedure of SAS, with Satterthwaite approximation to determine the denominator degrees of freedom for the test of fixed effects. Weight gain and average daily gain were tested for fixed effect of treatment using pen (treatment) and animal (pen) as random effects. All other variables were analyzed as repeated measures and tested for fixed effects of treatment, day, and treatment × day, using pen (treatment) and animal (pen) as random variables and pen (treatment) as subject. All results obtained on d 0 for each variable were included as covariates in each respective analysis, but were removed from the model when *P* >  0.10. The compound symmetric covariance structure was selected for BW and serum concentration of albumin and ROS and the first order autoregressive covariance structure was selected for all others variables. The covariance structures were selected according to the lowest Akaike information criterion. Means were separated using PDIFF and all results were reported as LSMEANS followed by SEM. A simple Pearson's correlation was calculated among the antioxidant variables using CORR procedure of SAS to determine the interrelation between these parameters. Significance was defined when *P* ≤ 0.05, and tendency when *P* >  0.05 and ≤ 0.10.

## 3 Results

### 3.1 Growth performance

The T4 lambs had greater BW on d 60, and T4 and T8 had greater BW on d 75, compared to T0 lambs (*P* =  0.05; Table 2). T4 and T8 lambs had greater weight gain and ADG from d 45–60 and from d 0–75 (*P* ≤  0.05; Table 2).

**Table 2:** Growth performance of lambs supplemented with biocholine concentrate during the reared period.

Variables	Treatments^a^			SEM	P-value	
	T0	T4	T8		Treat	Treat × day
Body weight, kg					0.37	0.05
d 0	29.13	29.15	29.16	0.51	–	–
d 15	33.27	33.40	32.86	0.51	–	–
d 30	36.60	37.03	36.65	0.51	–	–
d 45	40.46	41.15	40.90	0.51	–	–
d 60	43.51^b^	45.33^a^	44.75^ab^	0.51	–	–
d 75	45.93^b^	48.21^a^	48.55^a^	0.51	–	–
Weight gain, kg						
d 0 to 15	4.14	4.25	3.70	0.33	0.47	–
d 15 to 30	3.33	3.62	3.79	0.39	0.70	–
d 30 to 45	3.87	4.12	4.25	0.38	0.77	–
d 45–60	3.05^b^	4.18^a^	3.84^a^	0.33	0.05	–
d 60–75	2.42^b^	2.88^ab^	3.80^a^	0.41	0.05	–
d 0–75	16.80^b^	19.06^a^	19.38^a^	0.81	0.04	–
Average daily gain, kg/d						
d 0 to 15	0.27	0.28	0.25	0.02	0.48	–
d 15 to 30	0.22	0.24	0.25	0.03	0.70	–
d 30 to 45	0.26	0.28	0.28	0.03	0.76	–
d 45–60	0.20^b^	0.28^a^	0.26^a^	0.02	0.05	–
d 60–75	0.16^b^	0.19^ab^	0.25^a^	0.03	0.05	–
d 0–75	0.22^b^	0.25^a^	0.26^a^	0.01	0.04	–

^a−b^ Within a row, means without a common superscript differ (*P* ≤  0.05).

a The treatments T0, T4, and T8 represents 0, 4, and 8 g of biocholine per animal/day, respectively.

### 3.2 Rumen variables

No effects of BP were detected for rumen pH, acetate, total VFA and acetate/propionate ratio (*P* ≥  0.11; Table 3). However, T4 and T8 lambs had greater MBT activities compared to T0 lambs, and T8 lambs had greater MBT activities compared to T4 lambs (*P* =  0.01; Table 3). BP reduced the ruminal concentration of propionate at the beginning of the study (T4 and T8) but increased it (T8) at the end of the study (*P* =  0.01; Table 3). The T8 lambs had lower ruminal concentration of butyrate compared to T0 and T4 lambs (*P* =  0.01; Table 3).

**Table 3:** Rumen variables of lambs supplemented with biocholine concentrate during the reared period.

Variables^a^	Treatments^b^			SEM	P-value	
	T0	T4	T8		Treat	Treat × day
pH	6.27	6.24	6.33	0.08	0.75	0.11
MBT (minutes)	6.80^c^	8.44^b^	10.22^a^	0.48	0.01	0.71
*Volatile fatty acids (VFA)*						
Acetate (mmol/100 mL)	19.74	19.70	18.79	1.30	0.84	0.12
Propionate (mmol/100 mL)					0.28	0.01
d 15	9.84^a^	7.55^b^	6.95^b^	0.72	–	–
d 45	8.07^a^	6.05^b^	7.86^ab^	0.72	–	–
d 75	4.80^b^	6.50^ab^	7.74^a^	0.72	–	–
Butyrate(mmol/100 mL)	3.73^a^	3.68^a^	2.59^b^	0.44	0.01	0.11
Total VFA (mmol/100 mL)	31.04	30.08	28.89	1.62	0.65	0.13
Acetate/propionate ratio	2.73	3.01	2.50	0.19	0.19	0.40

^a−b^ Within a row, means without a common superscript differ (*P* ≤  0.05).

a Methylene blue test (MBT – indicate the microbiological activity).

b The treatments T0, T4, and T8 represents 0, 4, and 8 g of biocholine per animal/day, respectively.

### 3.3 Serum biochemistry: liver function and carbohydrate and protein metabolism

No effects of BP were detected for serum concentration of total protein, albumin, globulin, urea, AST, ALT and GGT (*P* ≥  0.27; Table 4). However, T4 lambs to have lower serum concentration of glucose, compared to T0 lambs (*P* =  0.05; Table 4).

**Table 4:** Serum biochemistry of lambs supplemented with biocholine concentrate during the reared period.

Variables^a^	Treatments^b^			SEM	*P*-value	
	T0	T4	T8		Treat	Treat × day
Total protein (g/dL)	6.62	6.90	7.00	0.30	0.64	0.85
Albumin (g/dL)	2.76	2.73	2.74	0.09	0.97	0.82
Globulin (g/dL)	3.67	4.44	4.20	0.35	0.12	0.81
Glucose (mg/dL)	81.17^x^	64.83^y^	70.78^xy^	5.29	0.05	0.19
Urea (mg/dL)	32.28	33.99	3379	2.25	0.84	0.57
AST (U/L)	117.32	129.88	114.64	6.80	0.27	0.88
ALT (U/L)	17.79	16.36	17.60	1.12	0.58	0.59
GGT (U/L)	97.88	102.03	87.49	5.18	0.16	0.91

^x−y^Within a row, means without a common superscript tended to differ (0.05 > *P* ≤  0.10).

a Aspartate aminotransferase (AST), alanine aminotransferase (ALT) and gama glutamil transferase (GGT).

b The treatments T0, T4, and T8 represents 0, 4, and 8 g of biocholine per animal/day, respectively.

### 3.4 Serum oxidant and antioxidant status

The T8 lambs had greater serum activity of SOD on d 15, T4 and T8 lambs on d 60, and T4 lambs on d 75, compared to T0 lambs (*P* =  0.02; Table 5). However, T4 and T8 lambs had greater serum activity of GST, compared to T0 lambs (*P* =  0.05; Table 5). Further, T8 lambs had lower serum levels of ROS, compared to T0 and T4 lambs (*P* =  0.05; Table 5).

**Table 5:** Serum oxidant/antioxidant variables of lambs supplemented with biocholine concentrate during the reared period.

Variables^a^	Treatments^b^			SEM	*P*-value	
	T0	T4	T8		Treat	Treat × day
SOD (U SOD/mg protein)					0.93	**0.02**
d 0	11.75	10.84	12.99	1.41	–	–
d 15	8.22^b^	7.85^b^	12.32^a^	1.41	–	–
d 30	10.92	10.89	10.40	1.41	–	–
d 45	8.75	5.98	6.55	1.41	–	–
d 60	2.67^b^	5.41^a^	5.01^a^	1.41	–	–
d 75	3.99^b^	5.52^a^	5.09^ab^	1.41	–	–
GST (μmol CDNB/min/mg protein)	2.25^y^	3.10^x^	3.03^x^	0.18	**0.05**	0.27
ROS (U DCF/mg of protein)	26.37^a^	25.98^a^	21.16^b^	1.59	**0.05**	0.11

^a−b^Within a row, means without a common superscript differ (*P* ≤  0.05).

^x−y^Within a row, means without a common superscript tended to differ (0.05 > *P* ≤  0.10).

a Superoxide dismutase (SOD), glutathione S-transferase (GST) and reactive oxygen species (ROS).

b The treatments T0, T4, and T8 represents 0, 4, and 8 g of biocholine per animal/day, respectively.

Negative Pearson's correlation coefficients were calculated between serum concentration of SOD and ROS or between serum concentration of GST and ROS (*P* ≤  0.05; Table 6); however, no effects were detected between SOD and GST (*P* =  0.25; Table 6).

**Table 6:** Pearson correlation coefficients among serum antioxidant variables.

Variables^a^	Pearson correlation coefficients	*P* - value
SOD × ROS	−0.20	0.04
GST × ROS	−0.18	0.05
SOD × GST	0.11	0.25

a Superoxide dismutase (SOD), glutathione transferase (GST) and reactive oxygen species (ROS).

## 4 Discussion

The BP intake increased the growth performance in the current study. Pinotti et al. [29] evaluated growth performance of beef cattle and found that supplementation with ruminally-protected choline increased body weight and ADG on d 89 of experiment. Similarly, Bryant et al. [30] found better growth performance of ruminally-protected choline supplemented steers and lambs and related to changes in lipid metabolism and metabolic hormones responsible for fat metabolism. We believe that this positive effect on weight gain is a consequence of a set of beneficial effects on the health of ewe lambs when they consumed BP, a commercial product that contains phospholipids, including phosphatidylcholine, in addition to components such as fiber (81.5 %). Therefore, we want to make it clear to readers that, although the commercial product is marketed as a source of choline, the set of results observed in the present study must be attributed to the product BP. In this study, the lambs used were young (4 months of age); therefore, the greater BW in animals that consumed BP could also reflect in better health. In this case, heavier animals does not necessarily mean fatter animals.

In the current study, BP did not affect the ruminal pH. Other studies evaluating the effects of choline chloride supplementation on ruminal fermentation also did not find significant changes in ruminal pH [31,8]. Atkins et al. [8] evaluated the concentration of VFA and found a higher concentration of acetate in animals supplemented with a chemical form of choline, whereas the concentration of propionate and butyrate showed no changes with choline chloride supplementation. The authors explain that any effect attributed to choline supplementation depends on interactions of the molecule with ruminal fermentation; did not find any apparent effects, although they identified a higher rate of ruminal degradation in the supplemented animals. It is important to emphasize that in our study we used BP, a commercial product that has a low phosphatidylcholine concentration in its composition, as well as other components that may have a direct or indirect effect on our results. Researchers suggest that phosphatidylcholine has greater bioavailability for ruminants, with natural resistance to ruminal degradation, suggesting that choline is able to pass through the rumen [15,32,33]. These properties that may account for the differences between VFA levels in our study and others that used choline chloride. Importantly, phosphatidylcholine is present in only 10 % of bacterial membranes, playing a key role in symbiotic and pathogenic interactions [34].

Our results suggest that BP modifies the concentration of ruminal VFA, probably by changing the bacterial population. It is possible that BP improved the rumen environment or had some antimicrobial effects, impairing the development of some groups of bacteria and thus probably providing better environment for others groups develop in greater quantity. Because this is a preliminary study, it was not possible to determine the mechanisms of action of BP against ruminal microorganisms; studies will be conducted by our research group to verify possible interactions plant extracts and microbes.

Higher levels of propionic at the end of the experiment may explain the greater weight gain of the animals, which had not occurred in the initial phase of the study, when the levels were lower. A possible explanation would be related to the fixed dose of BP during the whole experiment provided to the animals (4 and 8 g day), because in the 75 days of study lambs ewe gained approximately 19 kg; which would show a substantial discrepancy if the dose supplied was transformed into kilogram of body weight/animal at the beginning of the experiment compared to the middle and end of the experiment. These data suggest to the authors that the initially used doses of 4 and 8 g per animal/day were not adequate, that is, the dose should be lower and identified in future studies.

The changes in molar proportions of VFAs with the use of BP reflects an increase in the efficiency of transforming the energy contained in the ingested feed, into energy contained in the VFAs available for absorption in the rumen. Theoretically, each hexose (672 Kcal mol^−1^) contained in the diet that was converted by ruminal fermentation into acetate, butyrate and propionate, produces 420, 524, and 724 Kcal mol^−1^, respectively [35]. Thus, as the BP increased the rumen concentration of propionate, the efficiency of the use of the energy of the diet was also increased, and it allowed improve in the weight gain of the lambs.

The higher concentration of ruminal choline is probably due to the presence of ciliated protozoa. Investigators reported that the only source of phosphatidylcholine that is resistant to ruminal degradation is incorporated into the membrane of these ciliated protozoa [16,36,37]. Because the MBT indicated that microbiological activity was lower in the supplemented groups, we might suggest that there was an increase in protozoan production. Hypothetically, the increase in circulating phosphatidylcholine would stimulate the growth of this group of protozoa, consequently decreasing the concentration of ruminal bacteria, because protozoa are capable of bacterial engulfment [38].

Choline has a fundamental role in lipid and glucose metabolism, and is involved in the synthesis of molecules responsible for the orientation and function of various intracellular signaling proteins [39,40]. The supplemented groups had lower glucose levels than those of the control group, suggesting that there was an increase in insulin production; this was found in Suffolk lambs supplemented with rumen-protected choline [30]. However, [5], when evaluating serum metabolic parameters of cows during the transition period, found increases in glucose values after calving. These findings suggest that, depending on the production phase or animal species, carbohydrate metabolism can be altered.

Variables of protein metabolism, including globulins, did not differ in our study. Similarly, ALT, AST, and GGT activities did not differ between treatments despite BP supplementation, contrary to our hypothesis that BP would have a hepatoprotective effect, and reduce the activity of these enzymes. Importantly, these enzymes are released when hepatocytes are damaged; according to Zeisel et al. [6], when there is choline deficiency in the organism, there are increased levels of this enzyme and other enzymatic markers such as the enzyme AST. According to Rama Rao et al. [41], liver fat levels are inversely related to dietary choline content. Authors reported decreased liver fat deposition; protected choline supplementation gave rise to a moderate negative energy balance, with efficient fat mobilization and appropriate liver function in cows during the transition period [42]. Effects on hepatic response parameters were expected when choline is used in animal diets, as results different to the current study were described by Koujalagi et al. [43] in dairy cows supplemented with biocholine. We believe that if the dose was calculated in g BP/kg body weight, we could have obtained different results, as well as an effect on the clinical biochemical serum variables.

Choline is a donor of methyl groups that form methionine, an amino acid that is fundamental for maintaining antioxidant cellular defense systems that prevent oxidative stress and apoptosis [7]. This agrees with the results found in our experiment on antioxidant enzymatic activity. SOD and GST activities were higher and ROS levels were lower in supplemented animals, suggesting greater capacity of the defense systems. Similar results were reported for the livers of healthy and aflatoxin-challenged Nile tilapia [14,44,45]. Koujalagi et al. [43] reported similar results for SOD and GST activity in biocholine-fed dairy cows who showed minimized oxidative stress. These results reinforce the notion that BP has an antioxidant effect.

Complementary studies should be performed to elucidate the mechanism of action of BP in the rumen, and other parameters need to be verified to confirm the effect of supplementation on VFA production so as to identify the optimal dose of administration for small ruminants. It is also important to compare BP to pure choline in future studies, so as to determine whether the positive effects would be the same.

## 5 Conclusion

Supplementation with BP improved weight gain, increased the microbial activities in the rumen, and reduced the ruminal concentration of propionate at the beginning of the study, but increased it at the end of the study. The ruminal concentration of butyrate was decreased by BP supplementation. Intake of BP cause decreased the serum concentration of glucose and improved the antioxidant status of ewe lambs. The greatest effects were observed for 8 g of BP/animal/day. The results suggest that BP can be a potential feed additive for growing lambs.

## Ethics committee

The project was approved by the Animal Research Ethics Committee of the State University of Santa Catarina, protocol number 8560130319.

## Declaration of Competing Interest

The authors declare no conflict of interest.
