# Supplementary material for: Effects of biocholine powder supplementation in ewe lambs: Growth, rumen fermentation, antioxidant status, and metabolism
Source: Biotechnol Rep (Amst). 2020 Dec 24;29:e00580. doi: 10.1016/j.btre.2020.e00580 (PMC7779734; doi:10.1016/j.btre.2020.e00580)
Supplement: Supplementary file 1 [file mmc1.docx]

**Supplementary Table 1**: Validation of the methodology used to quantify the volatile fatty acids (VFA) in ruminal fluid.

| **Items** | **Acetate** | **Propionate** | **Butyrate** |
| --- | --- | --- | --- |
| R^2^ | 0.9971 | 0.9959 | 0.9958 |
| Equation | y = 0.0261x - 0.0889 | y = 0.0486x - 0.0798 | y = 0.0747x - 0.0991 |
| Linear range (mmol 100 mL^-1^)^1^ | 4.13 - 86.13 | 3.55 - 70.92 | 2.93 - 58.56 |
| LOD (mmol 100 mL^-1^) | 2.15 | 1.18 | 1.15 |
| LOQ (mmol 100 mL^-1^) | 4.13 | 3.55 | 2.93 |
| Accuracy (%) | 90.3 | 84.8 | 83.7 |
| Repeatability (RSD) | 1.78 | 1.84 | 0.86 |
| ^1^The linear range, LOD - limit of detection and LOQ - limit of quantitation expressed as mmol of VFA for 100 mL of ruminal fluid. | | | |
